# Comparison of two DNA microarrays for detection of plasmid-mediated antimicrobial resistance and virulence factor genes in clinical isolates of Enterobacteriaceae and non-Enterobacteriaceae

**DOI:** 10.1016/j.ijantimicag.2010.02.011

**Published:** 2010-06

**Authors:** Fiona Walsh, Niamh M. Cooke, Stephen G. Smith, Gary P. Moran, Fiona J. Cooke, Alasdair Ivens, John Wain, Thomas R. Rogers

**Affiliations:** a Department of Clinical Microbiology, Sir Patrick Dun Translational Research Laboratory, School of Medicine, University of Dublin, Trinity College, St James's Hospital Campus, Dublin 8, Ireland; b Microbiology Research Unit, Division of Oral Biosciences, Dublin Dental School and Hospital, University of Dublin, Trinity College, Dublin 2, Ireland; c Wellcome Trust Sanger Institute, Wellcome Trust Genome Campus, Hinxton, Cambridge CB10 1SA, UK

**Keywords:** β-Lactamases, Quinolones, Virulence, Molecular detection

## Abstract

A DNA microarray was developed to detect plasmid-mediated antimicrobial resistance (AR) and virulence factor (VF) genes in clinical isolates of Enterobacteriaceae and non-Enterobacteriaceae. The array was validated with the following bacterial species: *Escherichiacoli* (*n* = 17); *Klebsiellapneumoniae* (*n* = 3); *Enterobacter* spp. (*n* = 6); *Acinetobacter* genospecies 3 (*n* = 1); *Acinetobacterbaumannii* (*n* = 1); *Pseudomonasaeruginosa* (*n* = 2); and *Stenotrophomonasmaltophilia* (*n* = 2). The AR gene profiles of these isolates were identified by polymerase chain reaction (PCR). The DNA microarray consisted of 155 and 133 AR and VF gene probes, respectively. Results were compared with the commercially available Identibac AMR-ve Array Tube. Hybridisation results indicated that there was excellent correlation between PCR and array results for AR and VF genes. Genes conferring resistance to each antibiotic class were identified by the DNA array. Unusual resistance genes were also identified, such as *bla*_SHV-5_ in a *bla*_OXA-23_-positive carbapenem-resistant *A. baumannii*. The phylogenetic group of each *E. coli* isolate was verified by the array. These data demonstrate that it is possible to screen simultaneously for all important classes of mobile AR and VF genes in Enterobacteriaceae and non-Enterobacteriaceae whilst also assigning a correct phylogenetic group to *E. coli* isolates. Therefore, it is feasible to test clinical Gram-negative bacteria for all known AR genes and to provide important information regarding pathogenicity simultaneously.

## 1 Introduction

Levels of antimicrobial resistance in some developed countries are reaching crisis point, severely limiting treatment options for an increasing number of nosocomially acquired infections [1]. Rapid and reliable identification on nosocomial Gram-negative pathogens, along with characterisation of their resistance and virulence mechanisms, are highly desirable for effective management of infections. Early diagnosis and appropriate antimicrobial treatment are essential to decrease the acquisition and spread of antimicrobial resistance (AR) and/or virulence factor (VF) genes in these pathogens and thus improve patient survival and reduce healthcare costs [2].

Identification and characterisation of the genes responsible for AR and virulence in Gram-negative pathogens have been typically limited to gene-specific multiplex polymerase chain reaction (PCR) and sequencing. However, these methods have many common drawbacks. They are both labour intensive and time consuming and thus are not ideal for routine use in microbiology laboratories. Moreover, they only screen for a relatively small number of bacterial determinants, thereby overlooking numerous other AR and VF genes that may be present. There is therefore a demand for practical and cost-effective diagnostic methods that rapidly and simultaneously detect all AR and VF genes in any given bacterial strain.

Introduction of DNA microarray technology offers a viable alternative. Oligonucleotide-based microarrays are generally considered to be more specific than PCR-based microarrays [3–7]. In this study, we developed a customised glass slide oligonucleotide microarray, called the enterobacterial resistance–virulence (ERV) array. Recently, a commercial microarray has become available, the Identibac AMR-ve™ Array Tube (Veterinary Laboratories Agency, Addlestone, UK). This is a microtube DNA array that detects up to 58 AR genes of clinical importance in *Salmonella* spp. and *Escherichia coli*. The aim of this study was to validate the capacity of the customised ERV array to identify a representative collection of nosocomial Gram-negative pathogens and to detect all transferable AR and VF genes in these strains. The capacity of the Identibac AMR-ve Array Tube to detect all AR genes in these strains was also investigated for comparison. Results of both microarray systems were validated by PCR amplification and, where necessary, by direct sequencing.

## 2 Materials and methods

### 2.1 Bacterial strains

This study employed 32 isolates, comprising 17 *E. coli*, 3 *Klebsiellapneumoniae*, 6 *Enterobacter* spp., 1 *Acinetobacter* genospecies 3 and 1 *Acinetobacterbaumannii*, 2 *Stenotrophomonasmaltophilia* and 2 *Pseudomonasaeruginosa*. Isolates were recovered from blood culture, sputum, swab and urine samples from patients at St James's Hospital (Dublin, Ireland) between 2004 and 2006.

### 2.2 PCR detection of resistance genes

Enterobacteriaceae isolates were screened for the presence of *bla*_TEM_, *bla*_SHV_, *bla*_OXA_, *bla*_CTX-M_, transferable *ampC*, *aac(6*′*)-Ib-cr* and *qnr* by PCR. *Pseudomonas aeruginosa* and *Acinetobacter* spp. isolates were screened for the presence of *bla*_VIM_, *bla*_IMP_, *bla*_SPM_ and *bla*_GIM_. *Acinetobacter* isolates were also screened for *bla*_OXA-23-like_, *bla*_OXA-24-like_, *bla*_OXA-51-like_ and *bla*_OXA-58_. Full-length PCR amplicons used for sequencing were generated using PCR primers or primers designed to flank the entire gene. Ten *E. coli* isolates from blood cultures were screened for 15 VFs associated with extraintestinal pathogenic *E. coli* pathogenesis by PCR. Multiplex PCR amplifications employing three markers (*chuA*, *yjaA* and TSPE4.C2) allowed classification of these *E. coli* isolates into phylogenetic groups (A, B1, B2 or D). All oligonucleotides employed in this study are available at http://www.medicine.tcd.ie/clinical_microbiology/research/oligos.php.

### 2.3 ERV oligonucleotide probe design and array construction

Generation of the custom ERV oligonucleotide probe-based array and selection criteria of the genes were as described by Cooke et al. [8]. All target genes used to generate oligonucleotide probes and their corresponding nucleotide accession numbers are documented in Supplementary Table 1.

### 2.4 Genomic DNA labelling and microarray hybridisation

Genomic DNA was extracted using the Archive Pure DNA Cell/Tissue Kit (5PRIME, Hamburg, Germany) and labelled with Cy3-dCTP using the BioPrime^®^ DNA Labelling System (Invitrogen–BioSciences Ltd., Dun Laoghaire, Ireland). After labelling, probes were purified and applied to the microarray slide as outlined previously [9]. Following incubation in a sealed humid chamber at 42 °C for 16–24 h, slides were washed twice in a series of wash steps, dried and scanned immediately [9].

### 2.5 Data analysis

DNA microarray slides were scanned using a GenePix^®^ 4000B scanner [Axon Instruments, MDS Analytical Technologies (GB) Ltd., Wokingham, UK]. A signal intensity value was quantified for each fluorescent DNA ‘spot’ and local background data on the microarray using the GenePix Pro 6.1 software package. Hybridisation was deemed positive when the median raw fluorescence intensity for each spot was >140 or when >50% of the pixels in the feature exhibited a raw fluorescence intensity greater than background levels plus two standard deviations. These values were selected based on analysis of known positive genes in control isolates. The raw data and flag values were exported to GeneSpring GX 7.3 (Agilent Technologies, Little Island, Cork, Ireland), where the median signal value from all viable replicate spots, per slide, was used for further analysis.

### 2.6 Identibac AMR-ve Array Tube

The Identibac AMR-ve Array Tube was recently validated to identify AR genes (*n* = 58) in Gram-negative bacteria, including *E. coli* and *Salmonella*[3]. Amplification reactions, hybridisation, washing and scanning of the array tube were performed according to the manufacturer's instructions. Data normalisation, using the *ihfA* control gene probe, and subsequent analysis was carried out using IconoClust Software (AT-Version; CLONDIAG GmbH, Jena, Germany). The mean signal value for three replicate spots per probe was used for analysis. Probes with intensity values of ≥0.4 were consider positive and those with intensity values of <0.3 were considered negative, whilst those between 0.3 and <0.4 were considered ambiguous.

## 3 Results

### 3.1 Detection and discrimination of antimicrobial resistance and virulence factor genes with the ERV array

DNA from all isolates hybridised with between 5 and 39 AR probes. The test group included isolates that were negative for all genes investigated by PCR or were positive for only one gene. The phenotypic resistance profile of all isolates is described in Table 1. DNA from *E. coli* isolates hybridised with between 10 and 80 of the VF probes. DNA from other isolates hybridised with between 1 and 33 VF probes. However, it must be noted that the VF probes were designed from *E. coli* and as such many would not necessarily be present in non-*E. coli* isolates.

### 3.2 Correlation between PCR and ERV array hybridisation data

A good correlation was observed between the two methods for *bla*_TEM_, *bla*_SHV_, *bla*_OXA_, *bla*_CMY_ and *aac(6*′*)-Ib-cr* AR genes (Tables 2a and 2b). The microarray was more sensitive in detecting *bla*_TEM_ in one *E. coli* isolate and *bla*_SHV_ in one *K. pneumoniae* isolate, which were negative by PCR. One *Enterobacter* isolate was positive for *bla*_OXA_ by PCR and negative by microarray, but the opposite was the case for one *E. coli* isolate.

*Acinetobacter* resistance genotypes showed 100% identity between the two methods in discrimination between *A. baumannii* and *Acinetobacter* genospecies 3 using the presence of *bla*_OXA-51_ as a positive marker for *A. baumannii* identification. Identification of *bla*_OXA-51_ in *A. baumannii* could also be used as a species identification marker. However, *bla*_OXA-27_ was also identified in both isolates of *Acinetobacter*. One *P. aeruginosa* isolate was positive by PCR for *bla*_VIM_ but was positive by microarray for *bla*_SPM_. There were discrepancies between the PCR and microarray results for the detection of *bla*_DHA_ in *K. pneumoniae* and *Enterobacter* spp.

Seven isolates were positive for *bla*_CTX-M_ by microarray (CTX-M-9, *n* = 4) and negative by PCR. Of these seven isolates, four were phenotypically confirmed as extended-spectrum β-lactamase (ESBL)-producers, a further two were resistant to cefotaxime and ceftazidime, and the final isolate was susceptible to both antimicrobials (Table 1). All isolates were negative for other ESBL genes. The *bla*_TEM_ genes identified in these isolates were *bla*_TEM-1_.

The *bla*_CMY_ gene was detected by the microarray and there were no false-positives. There was 100% correlation between the two methods with regards to *aac(6*′*)-Ib-cr*-positive isolates. Most of the additional isolates that were positive by microarray for *aac(6*′*)-Ib* were gentamicin-resistant. There was no correlation between the two methods for detecting *qnr* genes.

A good correlation was also observed between the two methods for the 10 *E. coli* isolates that were screened for VF genes (Table 2a). Of the fimbrial VFs screened for, there was a 100% correlation for *papA* (*n* = 5), *papG* allele II (*n* = 4) and *sfa*/*focDE* (*n* = 3). PCR was more sensitive in detecting *papG* allele III and *afa*/*draBC*, whilst the microarray was more sensitive in detecting *fimH*. Amongst the fimbrial VFs screened, *papG* allele I gave the most varying correlations between the two methods. It was detected in four *E. coli* isolates by microarray but not by PCR.

There was a 100% correlation between the two methods for all *E. coli* isolates that encoded *hlyA* (*n* = 4) and *cnf1* (*n* = 3) toxin genes, and also for *traT* (*n* = 5) and *ibeA* (*n* = 1) genes. The microarray was more sensitive in detecting *fyuA* and *iutA* siderophore genes compared with PCR (8 vs. 7 and 10 vs. 6 positives, respectively). Similarly, the *kpsMT* II capsule biosynthesis gene was detected in six *E. coli* isolates by microarray but in four by PCR.

Nine *E. coli* isolates displayed a 100% correlation between the two methods with regards to classification of phylogenetic group. The microarray detected TSPE4.C2 in the final isolate that was not detected by PCR. However, because this isolate was also positive for *chuA* and negative for *yjaA*, the presence or absence of TSPE4.C2 would not have an overall effect on the phylogenetic group of the isolate, since either way it would be classified as belonging to group D.

Whilst the number of VFs identified in the non-invasive *E. coli* is greatly reduced compared with the bloodstream isolates, the overall patterns of the main VFs within the *E. coli* isolated from urine, swab and sputa samples corresponds to those identified within the bloodstream isolates. The urine isolates belonged to the phylogenetic groups A and B2, the swab isolate to group B2 and the sputum isolate to group B1.

### 3.3 Identibac AMR-ve Array Tube

Eleven *E. coli*, two *K. pneumoniae* and five *Enterobacter* spp. isolates that were employed to validate the ERV array were subsequently analysed by the Identibac AMR-ve Array Tube for comparison.

In total, nine *E. coli* isolates, two *K. pneumoniae* and four *Enterobacter* spp. and their duplicates generated successful hybridisation results. The remaining isolates and their duplicates failed to generate successful hybridisation results and therefore were not included in further analysis. The Identibac array showed good correlation with PCR results for the detection of β-lactamases and plasmid-mediated quinolone resistance determinants, except for the detection of *aac(6*′*)-Ib-cr* genes. Overall, both of the microarray systems detected more AR genes compared with PCR amplification analysis (Supplementary Table 2).

## 4 Discussion

In this study, we studied two microarray systems compared with conventional multiplex PCR methods, which rapidly provide comprehensive information about the genotypic profile of clinical bacterial isolates.

The ERV array detected all *bla*_TEM_, *bla*_SHV_, *bla*_OXA_, *bla*_CMY-2_ and *aac(6*′*)-Ib-cr* genes in all isolates. It also successfully classified all 10 *E. coli* isolates into the correct phylogenetic group. Many recent studies, particularly directed at *E. coli* bloodstream isolates, have highlighted the importance of phylogenetic group analyses in providing key information on mechanisms of pathogenesis and levels of AR, and in highlighting strains with enhanced invasive potentials as well as aiding epidemiological studies [10–12]. The ERV array also correctly detected a high proportion of VFs, especially with probes that corresponded to the adhesin and toxin genes.

Validation of this ERV array is unique in that it also included non-Enterobacteriaceae. This demonstrates the lack of false-positive cross-hybridisation between the plasmid resistance genes on the array and any chromosomal genes in these bacteria. One of the major advantages of screening isolates for all known AR genes is the identification of genes that may be co-located or co-transferred on mobile elements. By screening the *Acinetobacter* spp. isolates in this manner, we identified the presence of *bla*_SHV-5_ and *aac(6*′*)-Ib* in both isolates. This is a similar resistance gene profile to an ESBL-producing *A. baumannii* in the USA that was susceptible only to colistin and rifampicin [13]. The difference is that the carbapenem resistance in the isolates from our study could be accounted for by the presence of *bla*_OXA-23_ carbapenemase genes in both isolates. The microarray also detected a wide variety of aminoglycoside, chloramphenicol, tetracycline and trimethoprim resistance genes in each species of Enterobacteriaceae. As expected, few of these resistance genes were detected in the non-Enterobacteriaceae.

The original validation study of the Identibac AMR-ve Array Tube system reported 98.8% correlation between microarray and PCR results, therefore verifying the high specificity of the microarray probes [3]. However, the array was not able to detect resistance genes in a number of isolates that were resistant to the aminoglycosides, β-lactams, streptomycin, tetracycline, trimethoprim and sulphonamides. Taking into account the number of discrepancies reported by Batchelor et al. [3], some of which were also observed in this study, it is possible that this array format needs to undergo more stringent quality control checks, as there appears to be a high degree of batch variability leading to misdetection of a number of AR genes.

In conclusion, the ERV array proved more successful than the Identibac AMR-ve Array Tube. It not only contains more AR probes but also a comprehensive range of VF probes. There was a better concordance between the genes detected by PCR, or with their phenotypic results where PCR had not been performed, and those detected by the ERV array hybridisations. Also, all ERV array hybridisations successfully generated genotypic data using this system, unlike the Identibac AMR-ve Array Tube. Another important feature of the ERV array is that it is a very flexible system. It allows for future addition of supplemental target genes for further AR and VF genes as they are identified and characterised. Similarly, it allows for future refinement by adding or removing existing probes to improve the range, sensitivity and specificity. Plasmid-mediated resistance transfer in non-Enterobacteriaceae is an important factor in the proliferation and spread of antibiotic resistance in hospitals. Most microarray studies to date have used *E. coli* and *Salmonella* spp. for validation. This study is the first to include non-Enterobacteriaceae for validation of the array.

If the ERV array were to be combined with methodologies to amplify pathogen DNA directly from patient blood, this would provide for a powerful and effective diagnostic tool. We are currently examining whether whole-genome amplification methodologies such as OmniPlex may be suited to this aspiration [14].

## Figures and Tables

**Table 1:** Resistance profile of clinical isolates.

Isolate	Resistance phenotype							
	AUG	CTX	CAZ	MER	ESBL	CIP	AMI	GEN
*Escherichia coli* 2004-1	S	S	S	S	–	S	S	S
*E. coli* 2004-8	S	S	S	S	–	S	S	S
*E. coli* 2004-23	R	S	S	S	–	R	S	S
*E. coli* 2004-49	R	S	S	S	–	R	S	S
*E. coli* 2004-69	R	I	R	S	NEG	R	S	S
*E. coli* 2004-62	R	S	S	S	–	S	S	S
*E. coli* 2005-1	S	S	S	S	–	S	S	S
*E. coli* 2005-13	R	S	R	S	NEG	S	S	R
*E. coli* 2005-19	R	S	S	S	–	R	S	S
*E. coli* 2005-72	I	S	S	S	–	S	S	S
*E. coli* 235663	R	R	R	S	POS	R	S	S
*E. coli* 237088	R	S	S	S	NEG	R	S	S
*E. coli* 238727	R	R	S	S	POS	R	S	S
*E. coli* 238728	R	R	S	S	POS	R	S	S
*E. coli* 14815	R	R	R	S	POS	R	S	R
*E. coli* 232227	R	R	R	S	POS	S	S	R
*E. coli* 28265	R	R	R	S	POS	R	S	R
*Klebsiella pneumoniae* 207.2	R	R	R	R	POS	R	S	R
*K. pneumoniae* 234933	R	S	R	S	NEG	R	S	S
*K. pneumoniae* 26752	R	S	R	S	NEG	S	S	S
*Enterobacter* spp. 15584	R	R	R	S	POS	R	S	R
*Enterobacter* spp. 235204	R	R	R	S	NEG	R	S	S
*Enterobacter* spp. 235797	R	R	R	S	POS	I	S	R
*Enterobacter* spp. 236508	R	R	R	S	POS	R	S	S
*Enterobacter* spp. 236878	R	R	R	S	POS	S	S	R
*Enterobacter* spp. 32475	R	R	R	S	NEG	S	S	S
*Acinetobacter* genospecies 3 103165	–	R	S	R	–	S	S	S
*Acinetobacter baumannii* 229437	–	R	R	R	–	R	R	R
*Pseudomonas aeruginosa* 3	–	–	–	R	–	R	S	R
*P. aeruginosa* 53	–	–	–	S	–	R	S	R
*Stenotrophomonas maltophilia* 15251	R	R	R	R	–	R	R	R
*S. maltophilia* 89847	R	R	R	R	–	S	R	R

AUG, Augmentin (amoxicillin/clavulanic acid); CTX, cefotaxime; CAZ, ceftazidime; MER, meropenem; ESBL, extended–spectrum β–lactamase; CIP, ciprofloxacin; AMI, amikacin; GEN, gentamicin; NEG, negative; POS, positive; R, resistant; S, sensitive; I, intermediate.

**Table 2a:** Comparison of the enterobacterial resistance–virulence (ERV) array hybridisation results and characterised polymerase chain reaction (PCR) profiles of *Escherichia coli* isolates^a^.

	*E. coli* 2004-1	*E. coli* 2004-8	*E. coli* 2004-23	*E. coli* 2004-49	*E. coli* 2004-69	*E. coli* 2004-62	*E. coli* 2005-1	*E. coli* 2005-13	*E. coli* 2005-19	*E. coli* 2005-72	*E. coli* 235663	*E. coli* 237088	*E. coli* 238727	*E. coli* 238728	*E. coli* 14815	*E. coli* 232227	*E. coli* 28265
Antimicrobial resistance gene element																	
*bla*_TEM_	P	P	P	P	P	P		P	P	P					P	P	P
	M	M	M	M	M	M		M	M	M		M			M	M	M
*bla*_SHV_							P										
							M										
*bla*_OXA_													P	P			
												M	M	M			
*bla*_DHA_													PM				
*bla*_CTX-M_											P		P	P			
									M		M		M	M	M		M
*bla*_CMY_								P									
								M									

Virulence factor gene element																	
*chuA*	P	P		P	P		P	P									
	M	M		M	M		M	M				M		M			M
*yjaA*		P	P			P	P	P	P	P							
		M	M			M	M	M	M	M	M	M		M	M	M	M
TSPE4.C2		P						P									
	M	M						M				M		M	M	M	M
*papA*	P	P			P		P			P							
	M	M			M		M			M		M					
*papG* allele I																	
		M					M	M		M				M			
*papG* allele II	P	P			P		P										
	M	M			M		M										
*papG* allele III							P										
*sfa/focDE*		P					P	P									
		M					M	M			M		M				
*afa/draBC*	P																
*fimH*	P	P		P	P	P	P	P	P	P							
	M	M	M	M	M	M	M	M	M	M	M	M		M	M	M	M
*hlyA*	P	P					P	P									
	M	M					M	M									
*cnf1*		P					P	P									
		M					M	M									
*fyuA*		P			P	P	P	P	P	P							
	M	M			M	M	M	M	M	M	M	M		M	M	M	M
*iutA*	P	P			P	P			P	P							
	M	M	M	M	M	M	M	M	M	M	M	M		M	M	M	M
*kpsMT* II	P	P			P			P									
	M	M		M	M		M	M				M				M	
*traT*	P	P			P		P			P							
	M	M			M		M			M	M	M		M	M	M	M
*ibeA*								P									
								M									

P, PCR-positive detection; M, microarray-positive detection.

a Only isolates with the suffix 2004 and 2005 were analysed by PCR for the virulence determinants.

**Table 2b:** Comparison of ERV array hybridisation results and characterised PCR profiles.

Antimicrobial resistance gene element	*Klebsiella pneumoniae* 207.2	*K. pneumoniae* 234933	*K. pneumoniae* 26752	*Enterobacter* spp. 15584	*Enterobacter* spp. 235204	*Enterobacter* spp. 235797	*Enterobacter* spp. 236508	*Enterobacter* spp. 236878	*Enterobacter* spp. 32475	*Acinetobacter* genospecies 3 103165	*Acinetobacter baumannii* 229437	*Pseudomonas aeruginosa* 3	*P. aeruginosa* 53
*bla*_TEM_	M												
*bla*_SHV_	P							P					
	M	M						M					
*bla*_OXA_		P			P		P			P (*bla*_OXA-23_)	P (*bla*_OXA-23,__-51_)		
		M					M			M (*bla*_OXA-23,__-27_)	M (*bla*_OXA-23,__-27,__-51_)		
*bla*_VIM_												P	
												M (*bla*_SPM_)	
*bla*_DHA_		P	P		P		P						
	M	M				M	M	M					
*bla*_CTX-M_	P												
	M				M	M	M		M				
*aac(6*′*)-Ib[-cr]*		P			P	P	P						
	M	M		M	M	M	M	M	M				
*qnr*						P		P					
	M		M		M		M		M				

P, PCR-positive detection; M, microarray-positive detection.

## Appendix A Supplementary data

Supplementary Table 1 Genes and source of oligonucleotides used to design the enterobacterial resistance–virulence (ERV) array.

Supplementary Table 2 All genes identified by polymerase chain reaction (P), the enterobacterial resistance–virulence (ERV) array (M) and Identibac AMR-ve Array Tube (V).
